# Direct stimulation of bone mass by increased GH signalling in the osteoblasts of *Socs2*^*−/−*^ mice

**DOI:** 10.1530/JOE-14-0292

**Published:** 2014-10

**Authors:** R Dobie, V E MacRae, C Huesa, R van't Hof, S F Ahmed, C Farquharson

**Affiliations:** 1 Division of Developmental Biology, The Roslin Institute and R(D)SVS, The University of Edinburgh, Easter Bush, Edinburgh, Midlothian, EH25 9RG, Scotland, UK; 1 Institute of Ageing and Chronic Disease, University of Liverpool, Daulby Street, Liverpool, L69 3GA, UK; 2 Developmental Endocrinology Research Group, School of Medicine, University of Glasgow, Yorkhill, Glasgow, G3 8SJ, Scotland, UK

**Keywords:** GH/IGF1, bone, SOCS2, osteoblast

## Abstract

The suppressor of cytokine signalling (*Socs2* ^*−/−*^)-knockout mouse is characterised by an overgrowth phenotype due to enhanced GH signalling. The objective of this study was to define the *Socs2* ^*−/−*^ bone phenotype and determine whether GH promotes bone mass via IGF1-dependent mechanisms. Despite no elevation in systemic IGF1 levels, increased body weight in 4-week-old *Socs2* ^*−/−*^ mice following GH treatment was associated with increased cortical bone area (Ct.Ar) (*P*<0.01). Furthermore, detailed bone analysis of male and female juvenile and adult *Socs2* ^*−/−*^ mice revealed an altered cortical and trabecular phenotype consistent with the known anabolic effects of GH. Indeed, male *Socs2* ^*−/−*^ mice had increased Ct.Ar (*P*<0.05) and thickness associated with increased strength. Despite this, there was no elevation in hepatic *Igf1* expression, suggesting that the anabolic bone phenotype was the result of increased local GH action. Mechanistic studies showed that in osteoblasts and bone of *Socs2* ^*−/−*^ mice, STAT5 phosphorylation was significantly increased in response to GH. Conversely, overexpression of SOCS2 decreased GH-induced STAT5 signalling. Although an increase in *Igf1* expression was observed in *Socs2* ^*−/−*^ osteoblasts following GH, it was not evident *in vivo*. *Igf1* expression levels were not elevated in response to GH in 4-week-old mice and no alterations in expression was observed in bone samples of 6-week-old *Socs2* ^*−/−*^ mice. These studies emphasise the critical role of SOCS2 in controlling the local GH anabolic bone effects. We provide compelling evidence implicating SOCS2 in the regulation of GH osteoblast signalling and ultimately bone accrual, which maybe via mechanisms that are independent of IGF1 production *in vivo*.

## Introduction

Growth hormone (GH) is a key regulator of postnatal skeletal development; however, its mode of action remains unclear (Slootweg 1993, Ohlsson *et al*. 1998). It is likely to involve both endocrine (systemic) actions via hepatic insulin-like growth factor 1 (IGF1) production and autocrine/paracrine (local) actions via the osteoblast GH-receptor (GHR). In addition, local GH actions may be indirect (IGF1 dependent) or direct (IGF1 independent) (Isgaard *et al*. 1986, 1989, Le Roith *et al*. 2001).

The intimate relationship between GH and IGF1 makes it difficult to deduce the relative contributions of systemic and locally derived IGF1 to bone accrual. While *Ghr* ^*−/−*^ mice have recognised changes in skeletal mass and architecture, these effects may be mediated through IGF1, as *Ghr* ^*−/−*^ mice have reduced systemic IGF1 levels (Sims *et al*. 2000, Sjogren *et al*. 2000). Global *Igf1* ^*−/−*^ and osteoblast-specific *Igf1r* ^*−/−*^ mice also have altered bone architecture and diminished osteoid mineralisation (Bikle *et al*. 2001, Zhang *et al*. 2002).

Alterations in systemic IGF1, through the use of transgenic mouse models, offer an insight into the endocrine actions of IGF1 on bone turnover. Decreased systemic IGF1 levels results in an alteration in cortical geometry, with minimal alteration in trabecular bone (Sjogren *et al*. 2002, Yakar *et al*. 2002). Overexpression of *Igf1* in hepatocytes results in supraphysiological IGF1 levels which is anabolic to cortical bone, and is able to substitute for skeletally derived IGF1 in global *Igf1* ^*−/−*^ mice (Elis *et al*. 2010). Interpretation of some of these observations is complex as there is a 4.5-fold increase in systemic GH levels in LID mice (Yakar *et al*. 2002). This may contribute to the bone phenotype, and mask the effects of low circulating IGF1 levels on bone mass in this and other mouse models (Yakar *et al*. 2009). Ideally, the *in vivo* study of the local effects of GH on cortical and trabecular bone formation would benefit from animal models, in which both circulating GH and IGF1 levels are normal. Studies with the GHR antagonist pegvisomant show that in a state of hepatic IGF1 insufficiency, GH protects the skeleton (Courtland *et al*. 2011). The targeted overexpression of *Igf1* to osteoblasts indicates that locally derived IGF1 is anabolic for bone (Zhao *et al*. 2000). Conversely, mice with a targeted ablation of *Igf1* in osteoblasts have a catabolic bone phenotype with no reports of decreased circulating IGF1 levels (Govoni *et al*. 2007).

The suppressor of cytokine signalling (SOCS) protein family consists of at least eight proteins, with SOCS1–3 recognised as inhibitors of GH/IGF1 signal. Basal levels of these proteins are low, but when stimulated by GH, expression levels rise quickly (SOCS1 and 3) or more gradually (SOCS2), forming a negative feedback loop (Tollet-Egnell *et al*. 1999). The absence of SOCS2 in mice results in an overgrowth phenotype (Metcalf *et al*. 2000). Firm evidence exists to show that SOCS2 acts on the GH pathway. The overgrowth phenotype is lost in *Socs2* ^−/−^; *Ghrhr* ^*lit/lit*^, and diminished in *Socs2* ^*−/−*^; *Stat5b* ^*−/−*^ double-knockout mice (Greenhalgh *et al*. 2005, Flores-Morales *et al*. 2006). The *Socs2^−/−^* mouse is characterised by its overgrowth skeletal phenotype despite normal serum GH and IGF1 levels (Metcalf *et al*. 2000, Greenhalgh *et al*. 2002, MacRae *et al*. 2009). Aside from GH, SOCS2 has also been shown to be upregulated by oestrogen, and it is possible that GH controls bone mass, architecture, and strength in a gender-specific manner due to the influence of oestrogen on SOCS2 expression (Leung *et al*. 2003).

These data confirm the importance of the direct anabolic effects of GH, and establish *Socs2* ^*−/−*^ mice as an informative model for studying the local effects of GH on osteoblast function and bone accretion. Whether GH promotes osteoblast functions via IGF1-dependent and/or independent mechanisms remains unresolved. This study therefore explored the mechanisms by which bone accretion is enhanced in male and female *Socs2* ^*−/−*^ mice; and in doing so investigate the mechanisms of local GH control on osteoblast function.

## Material and methods

### Mice

The *Socs2* ^*−/−*^ mice used in this study were generated as described previously (MacRae *et al*. 2009). For genotyping, tail or ear biopsied DNA was analysed by PCR for SOCS2 WT or the neocassette (*Socs2* ^*−/−*^) using primers (Eurofins MWG Operon, London, UK) (Supplementary Table 1, see section on supplementary data given at the end of this article). All animal experiments were approved by Roslin Institute's Animal Users Committee, and the animals were maintained in accordance with Home Office (UK) guidelines for the care and use of laboratory animals.

### GH treatment

From 14 days of age, male WT and *Socs2* ^*−/−*^ mice received a s.c. injection of recombinant human (rh) GH (3 mg/kg), twice daily for 14 days. The control animals received sterile water. The mice were weighed each day before the first GH injection. Three hours after final GH-injection, the mice were killed. Blood was immediately extracted by cardiac puncture. The left femurs and livers were dissected, and the femurs had their epiphysis and marrow removed. Both livers and femurs were snap frozen in liquid nitrogen and stored at −80 °C for RNA extraction. Right femurs were dissected and stored in water at −20 °C for micro-computed tomography (μCT) analysis.

In addition, male WT and *Socs2* ^*−/−*^ mice at 24 days of age were given a single i.p. injection of rhGH (3 mg/kg) or sterile water for 15 min, before being killed, and livers and tibiae extracted. The epiphysis was dissected from the tibiae, and marrow removed by centrifugation. The samples were snap frozen and stored at −80 °C for protein extraction.

### Calcein labelling and tissue collection of adult and juvenile mice

Male WT and *Socs2* ^*−/−*^ mice received s.c. injections of 10 mg/kg calcein (Sigma) in sodium bicarbonate solution. In juvenile mice (6 weeks of age), injections were given 9 and 2 days before being killed. In adult mice (17 weeks of age), injections were given 16 and 2 days before being killed. At the point of sacrifice, 6-week-old male and female WT and *Socs2* ^*−/−*^ mice had their liver and right femur dissected and treated as previously described for RNA analysis. Left tibiae were also dissected and stored as previously described for μCT. Right tibiae were dissected for tissue processing.

### IGF1 and IGFBP3 ELISA

Serum samples were prepared, and total IGF1 levels were assessed by ELISA according to the manufacturer's instructions (Quantikine, R&D Systems, Minneapolis, MN, USA). IGF1 assays included a step to dissociate the potentially interfering binding proteins from the ligand. IGF1 and IGFBP3 levels were also assessed in conditioned medium of osteoblast cultures (see below).

### Microcomputed tomography

Tibiae were scanned using a μCT system (Skyscan 1172 X-Ray microtomograph, Bruker Corporation, Kontich, Belgium) to evaluate trabecular architecture and cortical bone geometry. For trabecular scanning, high-resolution scans with an isotropic voxel size of 5 μm were acquired (60 kV, 0.5 mm aluminium filter, 0.6° rotation angle). The isotropic voxel size was changed to 10 μm for cortical analysis. Two images were averaged at each rotation angle. The scans were reconstructed using NRecon software (Bruker). For analysis, a 1 mm section of the metaphysis was taken for the analysis of trabecular bone, using the base of the growth plate as a standard reference point. A 500 μm section of the mid-shaft was taken for the analysis of cortical bone, using the articulation with the fibula as a standard reference point. CTAn software (Bruker) was used to analyse the appropriate parameters previously outlined (Bouxsein *et al*. 2010).

### Mechanical testing

A Lloyd LRX5 materials testing machine (Lloyd Instruments, West Sussex, UK) fitted with a 500N load cell was used to determine bone stiffness and the point of failure of tibiae. The span was fixed at 10 mm, and the cross-head was lowered at 1 mm/min. Data were recorded after every 0.2 mm change in deflection. Each bone was tested to failure, with failure points being identified as the point of maximum load from the load–extension curve. The maximum stiffness was defined as the maximum gradient of the rising portion of this curve. Both values were calculated from a polynomial curve fitted to the rising region of the load–extension curve in SigmaPlot (Huesa *et al*. 2011).

### Tissue processing and calcein labelling of tibia

Tibiae fixed overnight in 4% paraformaldehyde (PFA) were embedded in methyl methacrylate. The sections (5 μm) of the proximal tibia were cut and processed using standard procedures (Idris *et al*. 2009). The mineral apposition rate (MAR) was estimated at both the endosteal and periosteal surfaces at the mid-diaphysis of each tibia (Huesa *et al*. 2011).

### Osteoblast isolation and culture

Osteoblasts were isolated from the calvaria of 3 to 5-day-old WT and *Socs2* ^*−/−*^ mice (Zhu *et al*. 2011, Prideaux *et al*. 2012). The cells were pooled, centrifuged, and plated in αMEM containing 10% foetal bovine serum,and 0.5% gentamicin (both Invitrogen). The cells were expanded in flasks in a humidified atmosphere of 95% air/5% CO_2_, and maintained at 37 °C until 80–90% confluence. They were then plated at 10 000 cells/cm^2^ in multi-well plates. At confluence, the cells were incubated for 24 h in serum-free medium containing 0.1% BSA (Sigma). In keeping with previous studies, the cells were exposed to 500 ng/ml rhGH or 50 ng/ml rhIGF1 (both Bachem, Merseyside, UK) (DiGirolamo *et al*. 2007, Pass *et al*. 2012). Further modifications of this culture protocol are indicated in the results.

### RNA extraction and quantitative RT-PCR

RNA was extracted from liver and cultured osteoblast samples (from above) using an RNeasy Mini Kit (Qiagen Ltd). Bone samples were homogenised using a mortar and pestle, and RNA was extracted using TRIzol reagent (Invitrogen). RNA content was measured by absorbance at 260 nm, and quality by 260/280 ratios. RT was carried out as described previously (Farquharson *et al*. 1999). RT-qPCR was undertaken using a Stratagene Mx3000P real-time qPCR machine with MxPro software (Stratagene, Santa Clara, CA, USA). Each cDNA sample was normalised to housekeeping genes *Gapdh* or *Atp5b* (Primer Design, Southampton, UK). The reactions were carried out with the genes of interest, primers for *Igf1* and *Igfbp3* (Invitrogen), as well as *Socs1*, *Socs2* and *Socs3* (Supplementary Table 1).

### Protein extraction and western blotting

The cells and tissue were scraped and lysed in either RIPA buffer or Phospho-Safe lysis buffer (both Sigma), containing protease inhibitors (Roche). Protein content was determined using the DC protein assay (Bio-Rad Laboratories). The lysates were run on 3–8 or 7% Tris–Acetate gels (for STAT, AKT and ERK1/2 proteins) or 10% Bis–Tris gels (for SOCS protein) (Supplementary Table 2, see section on supplementary data given at the end of this article). Following transfer, nitrocellulose films were blocked in 5% BSA, and probed for 90 min (STAT, AKT and ERK1/2 proteins) or overnight (SOCS protein) with primary antibodies. The nitrocellulose was probed with peroxidase labelled secondary antibodies for a further 90 min. ECL detection reagents were used to visualise bands on hyperfilm (Amersham, GE Healthcare, Bucks, UK). Where necessary, nitrocellulose was stripped using Restore Plus Stripping Buffer (Perbio Science, Northumberland, UK). Where appropriate, densitometry of western blotting was measured on three independent samples using Image J. Data are presented as phosphorylated protein normalised to total protein.

### Immunofluorescence of cultured cells

Primary osteoblasts isolated from WT and *Socs2* ^*−/−*^ mice were grown on collagen-coated glass cover slips. Following exposure to rhGH (500 ng/ml) for 20 min, the cells were fixed in 4% PFA (15 min). Following a PBS wash, they were then permeabilised using methanol (5 min) at −20 °C. After PBS washing, blocking buffer (5% normal goat serum/0.3% triton X-100) was added for 60 min and the cells were probed with pSTAT5 primary antibody overnight and subsequently a fluorochrome labelled secondary antibody for 60 min (Supplementary Table 2). Glass cover slips were then mounted onto slides using ProLong Gold antifade reagent with DAPI (Invitrogen) and cells were viewed by confocal microscopy.

### SOCS2 overexpression

The pEF-FLAG-1/mSOCS2 (SOCS2 overexpression; SOCS2+) and pEF-FLAG-1 (control) plasmids were obtained from Prof. D Hilton. MC3T3-E1 osteoblast-like cells (ECACC, Salisbury, UK) were grown to 70% confluency in osteoblast maintenance medium (as mentioned earlier) and co-transfected with pEF-FLAG-1/mSOCS2 or pEF-FLAG-1 and PCDNA3.1(+) plasmids at a ratio of 5:1 using FuGene 6 (Roche) as previously described (Pass *et al*. 2012). Geneticin (Sigma)-resistant colonies were picked using cloning cylinders (Sigma); expanded, frozen and maintained at −160 °C.

### Gene expression profiling

Gene expression profiling for JAK/STAT target genes (84 target genes) was carried out using RT^2^ profiler PCR arrays (Qiagen). In accordance with previous studies, osteoblasts were challenged with rhGH (500 ng/ml) for 4 h to detect genes directly stimulated by STAT translocation to the nucleus, and not those downstream of secondary messengers (Greenhalgh *et al*. 2005, Govoni *et al*. 2006). RNA was extracted as described, and 1 μg RNA was reverse transcribed with the RT^2^ profiler PCR array first-strand synthesis assay, followed by real-time PCR with RT^2^ real-time PCR master mix SYBR green.

### Statistical analyses

Data were tested for normality and equal variance using SigmaPlot (v11.0) (Systat Software, Inc., London, UK). Direct comparison between two sets of data was made by Student's *t*-test. For multiple comparisons, data were analysed for statistical significance by 2-way ANOVA incorporating pairwise comparisons. Data are presented as mean±s.e.m. Values *P*<0.05 were considered significant.

## Results

### Increased growth and bone development in *Socs2* ^*−/−*^ mice

**Figure 1:**
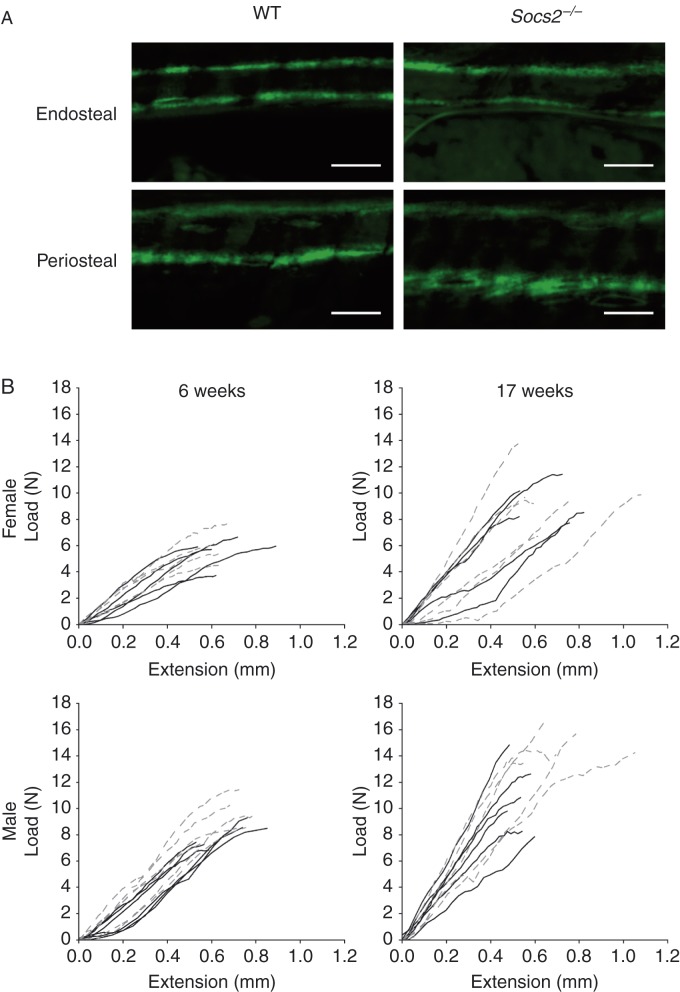
*Socs2* ^*−/−*^ cortical bone phenotype. (A) Images of periosteal and endosteal cortical bone apposition rate in tibia from juvenile 6-week-old male WT and *Socs2* ^*−/−*^ mice. Scale bar=25 μm. (B) Load vs extension curves to point of failure of tibia from juvenile 6-week-old and adult 17-week-old, male and female WT (black) and *Socs2* ^*−/−*^ (grey hatched) mice.

The increased growth rate of juvenile male *Socs2* ^*−/−*^ (103%; *P*<0.001), as measured by weight gain, was associated with increased periosteal expansion (Table 1 and Fig. 1A). Detailed analysis revealed increased periosteal MAR in the tibiae of male juvenile *Socs2* ^*−/−*^ mice (44%; *P*<0.01), but no change in endosteal MAR (Table 1 and Fig. 1A). The overgrowth phenotype was still apparent in adult male *Socs2* ^*−/−*^ mice (26% heavier; *P*<0.001); however, the growth rate was indistinguishable from age-matched WT mice (Table 1). At this point, double-labelled calcein was rarely observed in the tibia; suggesting minimal bone growth.

### Altered bone architecture and geometry in *Socs2* ^*−/−*^ mice

The bone volume/tissue volume of the proximal tibia was increased in all *Socs2* ^*−/−*^ mice (juvenile: male 34% (*P*<0.01), female 31% (*P*<0.01); adult: male 32% (*P*<0.05) female 24% (*P*<0.05). This was most likely due to the increased trabecular thickness noted in the *Socs2* ^*−/−*^ tibiae from juvenile (female 15% (*P*<0.01), male 14% (*P*<0.01)) and adult mice (female 39% (*P*<0.001), male 12% (*P*<0.01)). The structural model index (SMI) remained unchanged in the *Socs2* ^*−/−*^ tibia (Tables 1 and 2). Juvenile and adult, male and female *Socs2* ^*−/−*^ mice showed no alteration in trabecular BMD (Tables 1 and 2).

Analysis of cortical parameters revealed a 27% (*P*<0.001) and 28% (*P*<0.001) increase in cortical bone area (Ct.Ar) of the tibiae from juvenile and adult male *Socs2* ^*−/−*^ mice respectively. Similar changes were not noted in the equivalent *Socs2* ^*−/−*^ female mice (Tables 1 and 2). Grouping both ages together did reveal a genotype effect (*P*<0.05). Consistent with increased Ct.Ar, total tissue area (Tt.Ar) was significantly greater in all *Socs2* ^*−/−*^ tibia (*P*<0.05) (Tables 1 and 2). Increases in marrow space (Ma.Ar) were, however, far less marked, with only juvenile female (*P*<0.001) and adult male (*P*<0.001) *Socs2* ^*−/−*^ tibiae showing increased Ma.Ar. Analysis of male *Socs2* ^*−/−*^ tibiae, compared with WT, revealed a significant increase in cortical thickness (Ct.Th) (*P*<0.01). Age-defined analysis revealed that this increase was limited to juvenile *Socs2* ^*−/−*^ tibiae (*P*<0.01). Ct.Th was unchanged in female *Socs2* ^*−/−*^ tibiae (Tables 1 and 2). Polar moment of inertia was increased in the *Socs2* ^*−/−*^ tibiae of juvenile (female 37% (*P*<0.05), male 51% (*P*<0.05)) and adult mice (female 24% (*P*<0.05), male 82% (*P*<0.001)) (Tables 1 and 2). Cortical BMD in juvenile *Socs2* ^*−/−*^ and WT mice was similar; however, BMD was significantly decreased in adult male (*P*<0.05) and female (*P*<0.01) *Socs2* ^*−/−*^ mice (Tables 1 and 2).

### Increased strength of male *Socs2* ^*−/−*^ tibiae

Failure load, work to failure, load to maximum stiffness and maximum stiffness were unaltered in female *Socs2* ^*−/−*^ mice (Fig. 1B and Table 2). In contrast, tibiae from juvenile male *Socs2* ^*−/−*^ mice showed a 41% increase in work to failure (*P*<0.05). In adult mice, failure load (32%, *P*<0.05) and work to failure (90%, *P*<0.05) were both increased (Fig. 1B and Table 1). Load to maximum stiffness, and maximum stiffness of tibiae from male *Socs2* ^*−/−*^ mice were not altered at either age (Table 1).

### SOCS2 regulates bone growth responses to GH, independent of elevated systemic IGF1 levels

**Figure 2:**
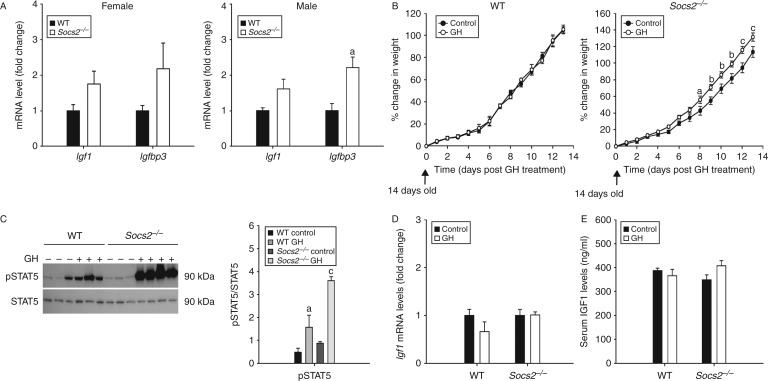
*SOCS2* regulation of systemic IGF1. (A) Transcript analysis of *Igf1* and *Igfbp3* in liver samples of 6-week-old male and female WT and *Socs2* ^*−/−*^ mice. Data represented relative to sex-matched WT as mean±s.e.m. (*n*=5). Significance from WT samples denoted by ^a^ *P*<0.05. (B) Graphs showing WT and *Socs2* ^*−/−*^ weight gain in response to twice daily GH (3 mg/kg) from 14 to 27 days of age. Data presented as mean±s.e.m. (*n*≥5). Significance from age-matched WT denoted by ^a^ *P*<0.05, ^b^ *P*<0.01, ^c^ *P*<0.001. (C) Western blotting analysis of phosphorylated (P−) STAT5 in 24-day-old WT (−GH (*n*=3); +GH (*n*=3)) and *Socs2* ^*−/−*^ (−GH (*n*=3); +GH (*n*=4)) liver samples following 15 min GH (3 mg/kg) treatment. Band intensities were quantified by densitometry and analysed for significance, as depicted in the graphs. Data presented as mean±s.e.m. (*n*≥3). Significance denoted by ^a^ *P*<0.05, ^c^ *P*<0.001. (D) Transcript analysis of *Igf1* in liver samples from 27-day-old WT and *Socs2* ^*−/−*^ mice following 14 days twice daily GH (3 mg/kg) treatment. Data presented relative to untreated samples as mean±s.e.m. (*n*=5). (E) Protein analysis by ELISA of IGF1 levels in serum extracted from 27-day-old WT and *Socs2* ^*−/−*^ mice following 14 days twice daily GH (3 mg/kg) treatment.

Comparison of juvenile WT and *Socs2* ^*−/−*^ mice revealed that despite the overgrowth phenotype of male (23%; *P*<0.001) and female (18%; *P*<0.01) *Socs2* ^*−/−*^ mice (Tables 1 and 2), transcript levels of hepatic *Igf1* were normal (Fig. 2A). Interestingly, there was a 2.2-fold increase in *Igfbp3* transcript levels in the livers of male *Socs2* ^*−/−*^ mice (*P*<0.05) (Fig. 2A).

To further assess the importance of systemic IGF1 in mediating the actions of GH on bone development in *Socs2* ^*−/−*^ mice, male WT and *Socs2* ^*−/−*^ mice were administered GH (Fig. 2B). Male *Socs2* ^*−/−*^ mice treated with GH showed increased growth following 8 days treatment (*P*<0.05) (Fig. 2B). WT mice showed no growth response at any time-point studied (Fig. 2B). Increased body weight in *Socs2* ^*−/−*^ mice was associated with an anabolic bone phenotype, characterised by increased Ct.Ar (20%, *P*<0.01) and polar moment of inertia (43%, *P*<0.05) (Table 3). No differences were observed in WT bone (Table 3). Liver samples extracted at day 24 (representing a stage of GH-induced growth promotion in *Socs2* ^*−/−*^ mice) revealed elevated pSTAT5 levels in the livers of WT (*P*<0.05) and *Socs2* ^*−/−*^ (*P*<0.001) mice following 15 min GH treatment. The levels of pSTAT5 were much higher in the liver of *Socs2* ^*−/−*^ mice (Fig. 2C). Despite increased STAT5 activation, increased growth and an anabolic bone phenotype (Fig. 2B, C and Table 3), GH treatment of *Socs2* ^*−/−*^ mice did not increase hepatic *Igf1* expression or systemic IGF1 levels at day 27 (Fig. 2D and E). Systemic IGF1 levels in GH-treated *Socs2* ^*−/−*^ mice remained similar to those observed for control and GH-treated WT mice (Fig. 2E). Taken together, these results emphasise that the overgrowth phenotype noted in *Socs2* ^*−/−*^ mice is a result of a non-systemic IGF1-mediated effects of local GH action.

### GH upregulates SOCS2 expression by osteoblasts

**Figure 3:**
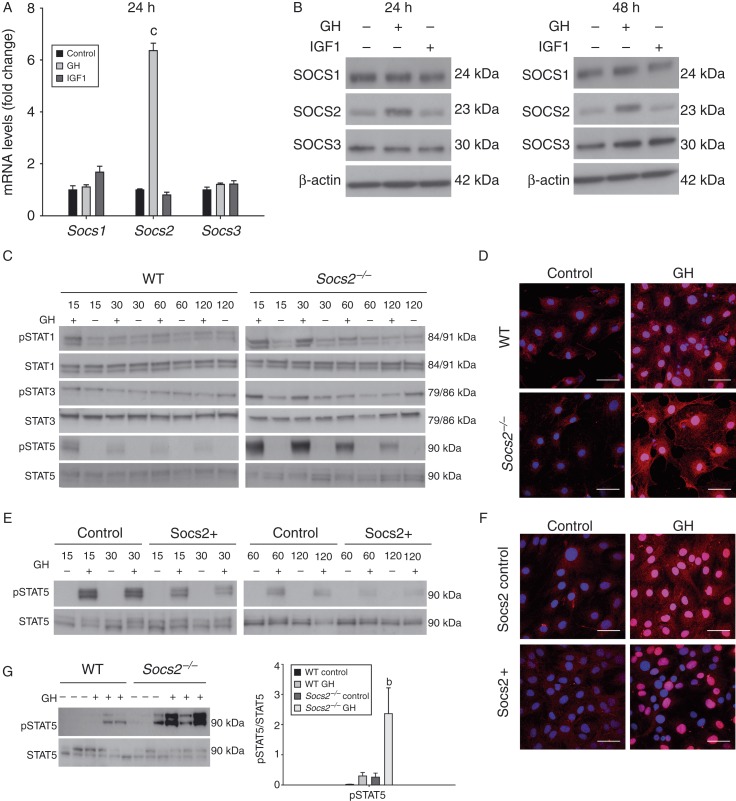
*SOCS2* regulation of GH-induced STAT signalling in osteoblasts and bone. (A) Transcript analysis of *Socs1*, *Socs2* and *Socs3* in WT osteoblasts following 24 h GH (500 ng/ml) or IGF1 (50 ng/ml) challenge. Transcript data represented relative to untreated samples as means±s.e.m. Significance denoted by ^c^ *P*<0.001. (B) Western blotting analysis of SOCS1, SOCS2 and SOCS3 in WT osteoblasts following 24 and 48 h GH (500 ng/ml) or IGF1 (50 ng/ml) treatment. (C) Western blotting analysis of phosphorylated (P−) STAT1, STAT3, and STAT5 in WT and *Socs2* ^*−/−*^ osteoblasts challenged with GH (500 ng/ml) for up to 120 min. (D) Immunofluorescence detection of pSTAT5 in WT and *Socs2* ^*−/−*^ osteoblasts following 20 min GH (500 ng/ml) challenge. (E) Analysis of (P−), STAT5 in SOCS2 overexpressing MC3T3 osteoblast-like cells and negative control-transfected cells following challenge with GH (500 ng/ml) for up to 120 min. (F) Immunofluorescence detection of pSTAT5 in SOCS2 overexpressing MC3T3 cells following 20 min GH (500 ng/ml) challenge. Scale bars for immunofluorescence represent 50 μm. Nucleus, blue; pSTAT5, pink. (G) Analysis of (P−) STAT5 in bone samples extracted from 24-day-old WT and *Socs2* ^*−/−*^ mice following 15 min GH (3 mg/kg) treatment. Band intensities were quantified by densitometry and analysed for significance, as depicted in the graphs. Data presented as mean±s.e.m. (*n*=3). Significance denoted by ^b^ *P*<0.01.

In WT osteoblasts, GH increased *Socs2*, but not *Socs1* and *Socs3*, mRNA expression after 24 h treatment (*P*<0.001) (Fig. 3A). This was confirmed and extended by western blotting analysis following 24 and 48 h GH treatment (Fig. 3B). IGF1 had no effects on SOCS1, SOCS2 and SOCS3 transcript or protein expression levels in WT cells (Fig. 3A and B). These data confirm SOCS2 as the primary SOCS protein regulating GH signalling in osteoblasts.

### SOCS2 negatively regulates GH-STAT intracellular signalling in osteoblasts and bone

**Figure 4:**
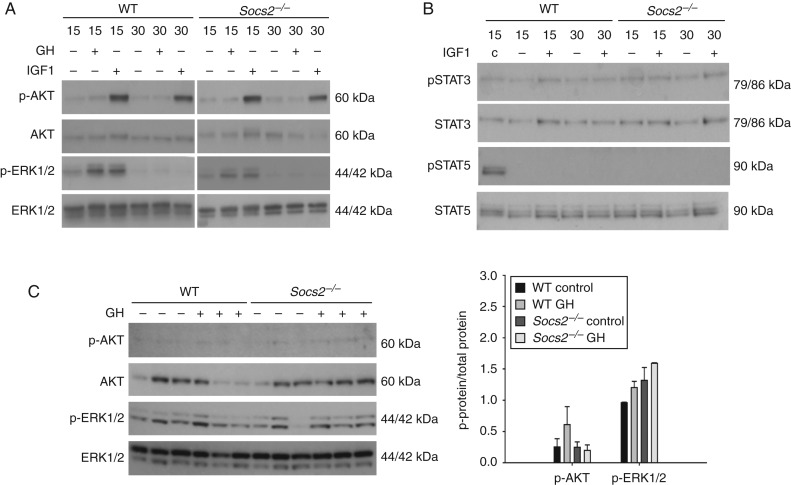
*SOCS2* regulation of GH or IGF1-induced AKT and ERK1/2 signalling. (A) Western blotting analysis of phosphorylated (P−) AKT and (P−) ERK1/2 in WT and *Socs2* ^*−/−*^ osteoblasts challenged with GH (500 ng/ml) or IGF1 (50 ng/ml) for up to 30 min. (B) Analysis of phosphorylated (P−) STAT3 and STAT5 in WT and *Socs2* ^*−/−*^ osteoblasts challenged with IGF1 (50 ng/ml) for up to 30 min. c, GH-treated WT osteoblast acting as a positive control. (C) Analysis of phosphorylated (P−) AKT and ERK1/2 in bone samples extracted from WT and *Socs2* ^*−/−*^ mice following 15 min GH (3 mg/kg) treatment. Band intensities were quantified by densitometry and analysed for significance, as depicted in the graphs. Data presented as mean±s.e.m. (*n*=3).

In WT osteoblasts, STAT3 was not activated (pSTAT) in response to GH, whereas pSTAT1 was only observed following 15 min of GH exposure. In contrast, pSTAT5 was observed at all-time points (15–120 min). Phosphorylation decreased with prolonged GH treatment. In *Socs2* ^*−/−*^ osteoblasts, the phosphorylation status of STAT1, STAT3 and STAT5 was both increased and prolonged by GH compared with WT (Fig. 3C). This was further confirmed by immunofluorescence where the stimulation of pSTAT5 by GH in both the cytoplasm and nucleus was much higher in *Socs2* ^*−/−*^ osteoblasts (Fig. 3D). Increased pSTAT5 levels were also observed in *Socs2* ^*−/−*^ bone samples following 15 min GH treatment (*P*<0.01) (Fig. 3G). The increase in pSTAT5 levels in bone from WT mice following GH treatment was far less, and did not reach significance from control bones (Fig. 3G). Comparable activation of ERK1/2 was observed in WT and *Socs2* ^*−/−*^ osteoblasts following GH treatment, whereas AKT activation was not observed (Fig. 4A). GH did not stimulate AKT or ERK1/2 activation in bone samples from WT or *Socs2* ^*−/−*^ mice following 15 min (Fig. 4C).

IGF1 did not phosphorylate STAT3 or STAT5, and the phosphorylation of AKT and ERK1/2 in response to IGF1 treatment was similar in WT and *Socs2* ^*−/−*^ osteoblasts (Fig. 4A and B). These data confirm the negative role of SOCS2 on GH-induced STAT signalling in osteoblasts and bone.

### Osteoblast SOCS2 overexpression inhibits GH-STAT intracellular signalling

To fully appreciate the inhibitory role of SOCS2 on GH-STAT5 signalling, the effects of SOCS2 overexpression in MC3T3 osteoblast-like cells were investigated (Fig. 3E and F). In comparison with control osteoblasts, STAT5 phosphorylation in response to GH (15–120 min) was less in SOCS2 overexpressing cells (Fig. 3E). These results were confirmed by immunofluorescence (Fig. 3F).

### Little evidence for local IGF1 regulation of increased bone mass in *Socs2* ^*−/−*^ mice

**Figure 5:**
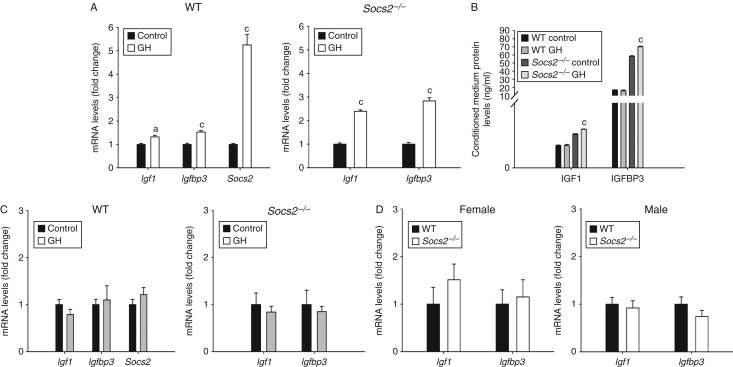
SOCS2 regulation of GH-induced IGF1 expression. (A) Transcript analysis of *Igf1*, *Igfbp3* and *Socs2* in WT and *Socs2* ^*−/−*^ osteoblasts following 48 h GH (500 ng/ml) treatment. (B) Protein analysis of IGF1 and IGFBP3 from conditioned medium from WT and *Socs2* ^*−/−*^ osteoblasts following 48 h GH (500 ng/ml) treatment. (C) Transcript analysis of *Igf1* and *Igfbp3* in bone samples extracted from 27-day-old male WT and *Socs2* ^−/−^ mice following 14 days twice daily GH (3 mg/kg) treatment. (D) Transcript analysis of *Igf1* and *Igfbp3* in bone samples extracted from 6-week-old male and female WT and *Socs2* ^−/−^ mice. All data represented as means±s.e.m. Significance from vehicle-treated samples denoted by ^a^ *P*<0.05, ^c^ *P*<0.001.

GH caused a small but significant increase in *Igf1* (1.32-fold, *P*<0.01) and *Igfbp3* (1.53-fold, *P*<0.001) expression in WT osteoblasts, whereas in *Socs2* ^*−/−*^ osteoblasts the increase in both *Igf1* (2.39-fold, *P*<0.001) and *Igf1bp3* (2.83-fold, *P*<0.001) expression was of a slightly greater magnitude (Fig. 5A). To further identify whether *Socs2* ^*−/−*^ osteoblasts had a greater responsiveness to GH, IGF1 and IGFBP3 protein levels were measured in the conditioned medium of cultured osteoblasts. In *Socs2* ^*−/−*^, the increase in IGF1 and IGFBP3 protein level following GH treatment, although significant, was extremely small (1.14-fold (*P*<0.001) and 1.20-fold (*P*<0.001) respectively). No significant changes in IGF1 and IGFBP3 protein levels were noted in WT osteoblasts (Fig. 5B). No increase in either *Igf1* or *Igfbp3* transcript levels was observed in tibia of WT and *Socs2* ^*−/−*^ mice administered GH (Fig. 5C).

To directly address the higher bone mass noted in the *Socs2* ^*−/−*^ mice, *Igf1* and *Igf1bp3* mRNA expression was quantified in femurs from mice previously analysed by μCT (Tables 1 and 2). The transcript levels of *Igf1* and *Igfbp3* in *Socs2* ^*−/−*^ and WT bones remained similar (Fig. 5D).

### Identification of osteoblast genes downstream of the JAK/STAT pathway stimulated by GH

In WT osteoblasts, the expression profiling revealed a significant induction of seven genes following 4 h GH treatment (*Sh2b2*, *Bcl2l1*, *Fr2*, *Fcgr1*, *Gata3*, *Gbp1* and *Socs2*). Only two genes showed over a twofold increase (*Fcgr1*, 4.46-fold, *P*<0.05 and *Socs2*, 4.55-fold, *P*<0.05) (Supplementary Figure 1 and Table 3, see section on supplementary data given at the end of this article). GH-treated *Socs2* ^*−/−*^ osteoblasts showed a significant increase in one gene (*Cdkn1a*) and a significant decrease in two genes (*Sla2* and *Stat4*), with Stat4 showing a greater than twofold change (−6.1-fold, *P*<0.05). In GH-treated *Socs2* ^*−/−*^ osteoblasts, *Fcgr1* increased by 3.7-fold, but the difference did not reach significance (*P*<0.076) (Supplementary Figure 1 and Table 3).

## Discussion

The overgrowth phenotype observed in *Socs2* ^*−/−*^ mice exhibits several features of deregulated GH signalling, including collagen accumulation in the dermis, and the decreased production of major urinary proteins (Metcalf *et al*. 2000, Greenhalgh *et al*. 2005). Furthermore, *Socs2* ^*−/−*^ mice are indistinguishable from WT littermates until 4–6 weeks, which coincides with the period of peak GH activity that occurs between postnatal days 20 and 40 (Wang *et al*. 2004). Circulating levels of GH and IGF1 in *Socs2* ^*−/−*^ mice are normal; however, it is not known whether this is a consequence of impaired hepatic GH signalling and downstream gene transcription. Given that STAT5b is important for upregulating IGF1 expression, and also essential for the *Socs2* ^*−/−*^ overgrowth phenotype, it is surprising that enhanced GH-induced hepatic STAT5 activation in *Socs2* ^*−/−*^ mice did not result in raised serum IGF1 levels (Davey *et al*. 1999, 2001, Greenhalgh *et al*. 2002). Interestingly, several studies have also reported no increase in basal systemic IGF1 levels in *Socs2* ^*−/−*^ mice. This suggests that the *Socs2* ^*−/−*^ overgrowth phenotype is a result of increased endogenous GH signalling at a local level (Metcalf *et al*. 2000, Lorentzon *et al*. 2005, MacRae *et al*. 2009). In agreement with this, the present study highlights that the increased growth observed in juvenile, male and female *Socs2* ^*−/−*^ mice was not associated with increased hepatic *Igf1* expression, which is recognised as the main source of systemic IGF1 (Sjogren *et al*. 1999, Yakar *et al*. 1999). Other GH-regulated genes such as *Igfbp3* are preferentially expressed in the *Socs2* ^*−/−*^ liver, and this was confirmed in male, but not in female mice (Rico-Bautista *et al*. 2005). The gender-specific effects of GH on *Igfbp3* expression have been noted previously (Bielohuby *et al*. 2011). These observations underline the importance of local GH signalling and confirm the value of the *Socs2* ^*−/−*^ mouse model in investigating the direct (IGF1 dependent or independent) anabolic effects of GH on growth and the skeleton.

In addition to their normal serum GH and IGF1 concentrations, *Socs2* ^*−/−*^ mice also have normal carbohydrate metabolism (*cf* *Alsko*, *Bp3ko*, *Lid* and *Igf1*-null mice) (Rico-Bautista *et al*. 2005, Yakar *et al*. 2009). The anabolic structural bone changes in *Gh*-transgenic mice are similar to those observed in *Socs2* ^*−/−*^ mice (Eckstein *et al*. 2004), but the *Gh*-transgenic mice suffer from insulin resistance, which is not observed in the *Socs2* ^*−/−*^ model (Olsson *et al*. 2005). Thus, the *Socs2* ^*−/−*^ mouse may represent an important model to study the pathways that promote bone accretion without leading to insulin resistance and altered carbohydrate metabolism.

The GH/IGF1 pathway is a critical regulator of osteoblast function, bone homeostasis and ultimately bone mass (Giustina *et al*. 2008). Global GH overexpression results in increased midshaft cross-sectional area; an observation that is more evident in males (Eckstein *et al*. 2004). Conversely, GH deficiency leads to decreased cortical periosteal circumference, cross-sectional area, and thickness; however, trabecular bone volume, number and thickness appear unchanged (Sims *et al*. 2000, Sjogren *et al*. 2000). Alterations in systemic IGF1 levels in these models make it difficult to delineate the relative contributions of GH and IGF1. Overexpression of GH in osteoblasts results in increased cortical area and strength, despite no increase in systemic IGF1 levels (Baker *et al*. 1992, Tseng *et al*. 1996). Conversely, overexpression of IGF1 in osteoblasts reveals an important role for local IGF1 in regulating trabecular architecture. There were minimal effects on cortical geometry, suggesting that all GH effects are not mediated through IGF1 (Zhao *et al*. 2000).

The present study found many similarities between the *Socs2* ^*−/−*^ and GH excess model, including increased Tt.Ar. This is consistent with previous bone phenotype analysis of the *Socs2* ^*−/−*^ mouse model, which focussed on 7-week-old female mice and the known anabolic effects of GH (MacRae *et al*. 2009). The increase in Tt.Ar is probably a result of increased periosteal bone formation. Juvenile male *Socs2* ^*−/−*^ mice had increased periosteal MAR, which is a commonly used parameter for the characterisation of bone formation (Schilling *et al*. 1992). Similar to the GH excess models, male *Socs2* ^*−/−*^ mice displayed a more pronounced anabolic phenotype, with increased Ct.Ar and Ct.Th providing greater support to the cortex and consequently increasing bone strength (Davison *et al*. 2006). The gender-specific differences may be a consequence of the reported regulatory role of oestrogen on SOCS2 expression; however, this relationship merits further investigation (Leung *et al*. 2003). The increased trabecular bone parameters observed in juvenile *Socs2* ^*−/−*^ mice may be secondary to altered growth plate organisation, which has been previously reported in similar aged mice (Pass *et al*. 2012). Increased trabecular bone is however still evident in adult mice when growth is negligible and the growth plate has been reduced to a thin layer of cells. This suggests that in adult mice, the trabecular bone phenotype is not secondary to alterations at the growth plate. Previous studies have produced varying results regarding the effects of SOCS2 on BMD, with some showing no effect, while others reporting a decrease (Lorentzon *et al*. 2005, MacRae *et al*. 2009). In this study, we report an age-dependent decrease in cortical BMD in *Socs2* ^*−/−*^ mice, with adults showing a decrease in BMD similar to that observed in the global GH excess model (Eckstein *et al*. 2004). Osteoblast-specific GH is anabolic to bone, and it has been proposed that this is at the expense of bone tissue integrity, such as increased cortical porosity which has been used as a predictor of BMD (Tseng *et al*. 1996, Wachter *et al*. 2002).

The activation of STAT1, STAT3 and STAT5 has been implicated in the regulation of gene transcription downstream of the GHR in various cell types; however in osteoblasts, the activation patterns remain unclear (Smit *et al*. 1996). In this study, we show that STAT1 and STAT5 are activated in response to GH in WT osteoblasts. In the absence of SOCS2, the GH-induced phosphorylation of osteoblast STAT1, STAT3 and STAT5, and the translocation of pSTAT5 to the osteoblast nucleus are increased compared with WT cells. The importance of SOCS2 in the regulation of GH-induced STAT5 activation in bone was further confirmed *in vivo*. The reliance of pSTAT5 for SOCS2 regulation of GH signalling has been clearly demonstrated in *Socs2* ^*−/−*^; *Stat5b* ^*−/−*^ double-knockout mice whose overgrowth phenotype is minimised (Greenhalgh *et al*. 2002). These data suggest that STAT 5 mediates GH anabolic actions on bone as reported for growth plate chondrocytes and linear bone growth (Teglund *et al*. 1998, Pass *et al*. 2012). Nevertheless, the bone phenotype of *Ghr* ^*−/−*^ mice is more severe than that of *Stat5ab* ^*−/−*^ mice, suggesting additional GH actions on osteoblasts that are STAT5 independent (Sims *et al*. 2000). While GH-induced phosphorylation of ERK1/2 in osteoblasts, this was not observed *in vivo*. Likewise, no evidence for a SOCS2 regulatory role of this pathway was found, proposing that the high *Socs2* ^*−/−*^ bone mass was not a result of increased GH-initiated ERK1/2 pathway.

Although the precise mechanism(s) by which SOCS2 negatively regulates GH signalling is unclear, the increased GH-induced phosphorylation of STAT1, STAT3 and STAT5 reported in osteoblasts implies that the inhibitory actions of SOCS2 are not STAT5 specific (Pass *et al*. 2009, Ahmed & Farquharson 2010). The observation that inhibition or deletion of SOCS2 does not modify GH-induced activation of the ERK1/2 and AKT pathways advocates that SOCS2 regulates STAT activation only. A role for SOCS1 and SOCS3 in negatively regulating the osteoblast STAT response to GH was not supported by our data and confirms our previous observations in growth plate chondrocytes (Pass *et al*. 2012).

The JAK/STAT pathway is required for GH-induced IGF1 production, and mice deficient in STAT5b show a decrease in serum IGF1 levels (Herrington *et al*. 2000, Sims *et al*. 2000). Therefore, in *Socs2* ^*−/−*^ osteoblasts and bone, the increased STAT phosphorylation in response to GH is likely to result in higher *Igf1* expression and thereby offer an explanation for the increased bone mass of *Socs2* ^*−/−*^ mice. Previous studies on cultured osteoblasts have reported that GH has either no effect or causes only a slight increase in *Igf1* expression (Schmid *et al*. 1994, DiGirolamo *et al*. 2007). This study reports similar small effects on *Igf1* and *Igfbp3* expression by GH, which were only slightly enhanced in *Socs2* ^*−/−*^ osteoblasts. This indicates that SOCS2 can limit GH's ability to stimulate osteoblast *Igf1* gene expression. Interestingly, it was also noted that basal levels of IGF1 and IGFBP3 were elevated in the condition medium of *Socs2* ^*−/−*^ osteoblasts. It is however unlikely that these effects are mediated via GH as osteoblasts were serum starved. Nevertheless, this observation warrants further investigation. Our *in vitro* data, obtained in a primary cell culture scenario, were however not confirmed in two separate *in vivo* models. GH administration to *Socs2* ^*−/−*^ mice was anabolic to bone, but showed no increase in bone *Igf1* and *Igfbp3* expression. Similarly, *Socs2* ^*−/−*^ mice with an over growth phenotype, increased bone mass and strength had unchanged levels of bone *Igf1* and *Igfbp3*. The attempts to identify alternative signalling pathways, downstream of STAT signalling that are both stimulated by GH and negatively controlled by SOCS2, were unrewarding. Further studies are required to identify the specific signalling pathways initiated by GH's direct effects on osteoblasts.

In conclusion, this study underscores the critical role of SOCS2 in controlling GH's anabolic effects on bone. It also provides compelling evidence to support the notion that GH can regulate osteoblast function and ultimately bone mass via local mechanisms, that *in vivo* are perhaps independent of IGF1 production.

## Supplementary data

This is linked to the online version of the paper at http://dx.doi.org/10.1530/JOE-14-0292.

Supplementary Data

## Figures and Tables

**Table 1:** Effects of SOCS2 on bone development in juvenile and adult male mice. Data are presented as mean±s.e.m. *n*=3–5 for growth data and *n*=6 for trabecular, cortical and biomechanical data

	**6 weeks**		**17 weeks**	
WT	*Socs2* ^*−/−*^	WT	*Socs2* ^*−/−*^
Growth				
Weight (g)	21.3±0.29	26.1±0.62^‡^	32.9±1.68	41.5±1.04^‡^
Weight gain (g/day)	0.29±0.034	0.59±0.030^‡^	0.02±0.030	0.02±0.024
Cortical endosteal MAR (μm/day)	3.3±0.15	3.9±0.07	NA	NA
Cortical periosteal MAR (μm/day)	3.6±0.30	5.1±0.25^†^	NA	NA
Trabecular				
BV/TV (%)	15.4±0.69	20.6±1.72^†^	14.9±1.9	19.6±1.07*
Tb.N (1/mm)	3.4±0.01	4.0±0.25	3.0±0.41	3.5±0.19
Tb.Th (mm)	0.04±0.001	0.05±0.002^†^	0.05±0.002	0.06±0.001^†^
Tb.Sp (mm)	0.17±0.01	0.16±0.01	0.2±0.01	0.18±0.01
SMI	1.9±0.05	1.7±0.08	1.7±0.18	1.5±0.05
BMD (g/cm^3^)	1.44±0.01	1.46±0.03	1.55±0.006	1.45±0.06
Cortical				
Tt.Ar (mm^2^)	0.8±0.03	1.0±0.03^†^	0.9±0.06	1.3±0.04^‡^
Ct.Ar (mm^2^)	0.6±0.01	0.7±0.03^‡^	0.7±0.03	0.9±0.02^‡^
Ma.Ar (mm^2^)	0.23±0.016	0.25±0.015	0.25±0.029	0.41±0.018^‡^
Ct.Th (mm)	0.25±0.008	0.28±0.011^†^	0.27±0.003	0.28±0.003
J (mm^4^)	0.10±0.003	0.15±0.012*	0.13±0.016	0.24±0.014^‡^
BMD (g/cm^3^)	1.31±0.016	1.29±0.04	1.42±0.002	1.35±0.008*
Biomechanical				
Failure load (N)	8.0±0.39	9.4±0.57	11.1±2.46	14.6±1.59*
Work to failure (mJ)	2.6±0.23	3.6±0.34*	3.3±0.71	6.2±2.35*
Load to maximum stiffness (N)	4.4±0.33	4.7±0.41	6.2±1.62	6.8±1.21
Maximum stiffness (N/mm)	18.6±1.52	19.6±3.18	28.4±8.57	30.3±10.57

For trabecular data BV/TV, bone volume/tissue volume; Tb.N, trabecular number; Tb.Th, trabecular thickness; Tb.Sp, trabecular separation; SMI, structural model index. For cortical data Tt.Ar, total tissue area; Ct.Ar, cortical bone area; Ma.Ar, medullary area; Ct.Th, cortical thickness; J, polar moment of inertia. Significance from age-matched WT mice is denoted by *P<0.05, ^†^ *P*<0.01, ^‡^ *P*<0.001.

**Table 2:** Effects of SOCS2 on bone development in juvenile and adult female mice. Data are presented as mean±s.e.m. (*n*=6)

	**6 weeks**		**17 weeks**	
WT	*Socs2* ^*−/−*^	WT	*Socs2* ^*−/−*^
Growth				
Weight (g)	16.3±0.62	19.3±0.4^†^	26.2±0.91	31.3±0.99^†^
Weight gain (g/day)	0.14±0.04	0.21±0.04	0.06±0.037	0.02±0.05
Trabecular				
BV/TV (%)	9.7±0.25	12.7±0.88^†^	8.0±0.38	9.9±0.52*
Tb.N (1/mm)	2.4±0.09	2.7±0.13*	1.6±0.10	1.4±0.09
Tb.Th (mm)	0.04±0.001	0.05±0.002^†^	0.05±0.002	0.07±0.001^‡^
Tb.Sp (mm)	0.22±0.01	0.23±0.01	0.28±0.01	0.33±0.03*
SMI	2.0±0.04	2.0±0.05	2.4±0.28	2.3±0.10
BMD (g/cm^3^)	1.48±0.006	1.47±0.01	1.56±0.01	1.57±0.01
Cortical				
Tt.Ar (mm^2^)	0.6±0.02	0.7±0.01^†^	0.7±0.02	0.8±0.04*
Ct.Ar (mm^2^)	0.4±0.01	0.5±0.01	0.6±0.004	0.6±0.02
Ma.Ar (mm^2^)	0.19±0.008	0.26±0.008^‡^	0.16±0.008	0.12±0.039
Ct.Th (mm)	0.21±0.005	0.20±0.002	0.26±0.009	0.27±0.007
J (mm^4^)	0.05±0.003	0.07±0.003*	0.08±0.007	0.09±0.007*
BMD (g/cm^3^)	1.34±0.007	1.32±0.006	1.44±0.005	1.41±0.006^†^
Biomechanical				
Failure load (N)	5.6±0.51	5.7±0.45	9.3±0.54	10.0±0.90
Work to failure (mJ)	2.4±0.31	2.0±0.22	3.0±0.27	3.0±0.26
Load to maximum stiffness (N)	2.7±0.35	2.1±0.27	5.5±0.8	5.7±0.5
Maximum stiffness (N/mm)	11.1±1.32	12.5±0.95	18.2±1.47	22.7±3.42

For trabecular data BV/TV, bone volume/tissue volume; Tb.N, trabecular number; Tb.Th, trabecular thickness; Tb.Sp, trabecular separation; SMI, structural model index. For cortical data Tt.Ar, total tissue area; Ct.Ar, cortical bone area; Ma.Ar, medullary area; Ct.Th, cortical thickness; J, polar moment of inertia. Significance from age-matched WT mice is denoted by *P<0.05, ^†^ *P*<0.01, ^‡^ *P*<0.001.

**Table 3:** Effects of SOCS2 on cortical bone development in male mice following 2 weeks rhGH (3 mg/kg) treatment. Data are presented as mean±s.e.m. (*n*≥4)

	**WT**		***Socs2^−/−^***	
Control	GH	Control	GH
Tt.Ar (mm^2^)	1.33±0.051	1.30±0.070	1.27±0.050	1.45±0.070
Ct.Ar (mm^2^)	0.45±0.023	0.43±0.023	0.41±0.009	0.49±0.017^†^
Ma.Ar (mm^2^)	0.88±0.032	0.87±0.048	0.86±0.050	0.96±0.061
Ct.Th (mm)	0.12±0.003	0.12±0.003	0.12±0.004	0.13±0.004
J (mm^4^)	0.17±.0.018	0.15±0.017	0.14±0.007	0.20±0.015*

For cortical data Tt.Ar, total tissue area; Ct.Ar, cortical bone area; Ma.Ar, medullary area; Ct.Th, cortical thickness; J, polar moment of inertia. Significance from genotype-matched control group is denoted by *P<0.05, ^†^ *P*<0.01.
