# Supplementary material for: Direct stimulation of bone mass by increased GH signalling in the osteoblasts of Socs2−/− mice
Source: J Endocrinol. 2014 Jul 29;223(1):93–106. doi: 10.1530/JOE-14-0292 (PMC4166176; doi:10.1530/JOE-14-0292)
Supplement: Supplementary Data [file supp_223_1_93__index.html]

Direct stimulation of bone mass by increased GH signalling in the osteoblasts of Socs2−/− mice — SOCS2 regulation of local GH action — Supplementary Data 

# Direct stimulation of bone mass by increased GH signalling in the osteoblasts of *Socs2**−/−* mice

## Supplementary Data

**Files in this Data Supplement:**

- Supplementary Figure 1 - (PDF 32 KB)
- Supplementary Table 1 - Primers used for genotyping and PCR analysis. (PDF 19 KB)
- Supplementary Table 2 - Primary antibodies and their dilutions used for western blotting and immunofluorescence. (PDF 32 KB)
- Supplementary Table 3 - Genes regulated by GH downstream of JAK/STAT in WT and *Socs2-/-* osteoblasts. (PDF 24 KB)
