# Supplementary material for: Direct stimulation of bone mass by increased GH signalling in the osteoblasts of Socs2−/− mice
Source: J Endocrinol. 2014 Jul 29;223(1):93–106. doi: 10.1530/JOE-14-0292 (PMC4166176; doi:10.1530/JOE-14-0292)
Supplement: Supplementary Data [file supp_JOE-14-0292_Supplementary_Figure_1.pdf]

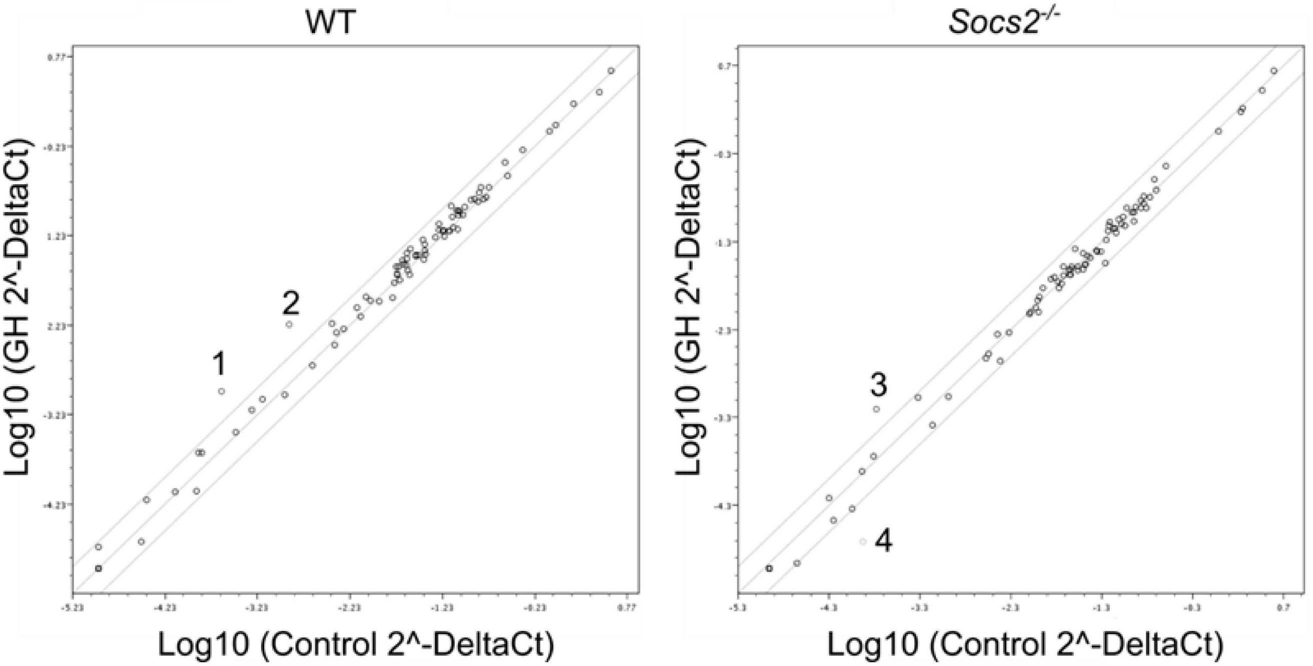

Supplementary Figure 1 Comparison of the normalised expression of genes related to the JAK/STAT pathway in WT and *Socs2*$^{-/-}$ osteoblasts following 4hrs GH treatment. Genes are plotted against one another to visualise gene expression changes. Boundaries indicate a fold change of 2. Numbered dots refer to genes listed in Supplementary Table 3.
