# Supplementary material for: Direct stimulation of bone mass by increased GH signalling in the osteoblasts of Socs2−/− mice
Source: J Endocrinol. 2014 Jul 29;223(1):93–106. doi: 10.1530/JOE-14-0292 (PMC4166176; doi:10.1530/JOE-14-0292)
Supplement: Supplementary Data [file supp_JOE-14-0292_Supplementary_table_1.pdf]

Supplementary Table 1. Primers used for genotyping and PCR analysis

	**Forward (5'-3')**	**Reverse (5'-3')**
Genotyping		
SOCS2	TGTTTGACTGAGCTCGCGC	CAACTTTAGTGTCTTGGATCT
Neo	ACCCTGCACACTCTCGTTTTG	CCTCGACTAAACACATGTAAAGC
PCR Analysis		
Socs1	TCCGATTACCGGCGCATCACG	CTCCAGCAGCTCGAAAAGGCA
Socs2	TGGCTGCTCAAGATCAAATG	TGTCCTCCTGGAAATGGAAG
Socs3	GAGTACCCCCAAGAGAGCTTACTA	CTCCTTAAAGTGGAGCATCATACTG
