# Supplementary material for: Direct stimulation of bone mass by increased GH signalling in the osteoblasts of Socs2−/− mice
Source: J Endocrinol. 2014 Jul 29;223(1):93–106. doi: 10.1530/JOE-14-0292 (PMC4166176; doi:10.1530/JOE-14-0292)
Supplement: Supplementary Data [file supp_JOE-14-0292_Supplementary_Table_2.pdf]

**Supplementary Table 2** Primary antibodies and their dilutions used for western blotting and immunofluorescence.

| **Peptide/protein target** | **Name of Antibody** | **Species raised in; monoclonal or polyclonal** | **Dilution used** |
|---|---|---|---|
| **SOCS1** | SOCS1 (ab) | goat polyclonal | 1 in 666 |
| **SOCS2** | SOCS2 (cs) | rabbit polyclonal | 1 in 1000 |
| **SOCS3** | SOCS3 (ab) | rabbit polyclonal | 1 in 369 |
| **β-Actin** | β-Actin (s) | mouse monoclonal | 1 in 25,000 |
| **P-STAT1** | P-STAT1 (cs) | rabbit polyclonal | 1 in 1000 |
| **STAT1** | STAT1 (cs) | rabbit polyclonal | 1 in 1000 |
| **P-STAT3** | P-STAT3 (cs) | rabbit polyclonal | 1 in 1000 |
| **STAT3** | STAT3 (cs) | rabbit polyclonal | 1 in 1000 |
| **P-STAT5** | P-STAT5 (cs) | rabbit polyclonal | 1 in 1000 |
| **STAT5** | STAT5 (cs) | rabbit polyclonal | 1 in 1000 |
| **P-STAT 5** | P-STAT5 (tyr 694) (D47E7) (cs) (IF) | rabbit monoclonal | 1 in 50 |
| **P-AKT** | P-AKT (cs) | rabbit polyclonal | 1 in 1000 |
| **AKT** | AKT (cs) | rabbit polyclonal | 1 in 1000 |
| **P-P44/42** | P-P44/42 MAPK (ERK1/2) (cs) | rabbit polyclonal | 1 in 1000 |
| **P44/42** | P44/42 MAPK (ERK1/2) (cs) | rabbit polyclonal | 1 in 1000 |

cs = Cell Signalling Technology (New England Biolabs, Hitchin, UK); s = Sigma; ab = Abcam (Cambridge, UK). (IF) = for immunofluorescence
