# Supplementary material for: Direct stimulation of bone mass by increased GH signalling in the osteoblasts of Socs2−/− mice
Source: J Endocrinol. 2014 Jul 29;223(1):93–106. doi: 10.1530/JOE-14-0292 (PMC4166176; doi:10.1530/JOE-14-0292)
Supplement: Supplementary Data [file supp_JOE-14-0292_Supplementary_table_3.pdf]

Supplementary Table 3. Genes regulated by GH downstream of JAK/STAT in WT and *Socs2*$^{-/-}$ osteoblasts

WT					*Socs2*$^{-/-}$				
Gene Symbol	Number	Ref. Seq.	Fold Change	p value	Gene Symbol	Number	Ref. Seq.	Fold Change	p value
Sh2b2		NM_018825	1.33	0.025	*Cdkn1a*		NM_007669	1.42	0.033
Bcl2l1		NM_009743	1.45	0.010	*Fcgr1*	3	NM_010186	3.71	0.076
Fr2		NM_010169	1.35	0.023	*Sla2*		NM_029983	-1.66	0.034
Gata3		NM_008091	1.45	0.020	*Stat4*	4	NM_011487	-6.08	0.023
Gbp1		NM_010259	1.72	0.003					
Socs2	1	NM_007706	4.55	0.020					
Fcgr1	2	NM_010186	4.46	0.034					

Numbers refer to dots on Supplementary Figure 1.
